# Supplementary material for: HIVprotI: an integrated web based platform for prediction and design of HIV proteins inhibitors
Source: J Cheminform. 2018 Mar 9;10:12. doi: 10.1186/s13321-018-0266-y (PMC5845081; doi:10.1186/s13321-018-0266-y)
Supplement: Supplementary file 2 — Additional file 2. Source code of HIVProtI web server. [file 13321_2018_266_MOESM2_ESM.zip › HIVprotI_Source-code/hivprotI/weka-3-6-6/doc/weka/gui/beans/FlowRunner.html]

FlowRunner


---


|  |  |  |
| --- | --- | --- |
| **Overview** \| **Package** \| **Class** \| **Tree** \| **Deprecated** \| **Index** \| **Help** \| **Weka's home** | |  |
| **PREV CLASS**   **NEXT CLASS** | **FRAMES**    **NO FRAMES**     **All Classes** |  |
| SUMMARY: NESTED \| FIELD \| CONSTR \| METHOD | DETAIL: FIELD \| CONSTR \| METHOD |  |


---


## weka.gui.beans Class FlowRunner

```
java.lang.Object
  weka.gui.beans.FlowRunner
```

**All Implemented Interfaces:**: RevisionHandler

---

``` public class FlowRunner extends java.lang.Object implements RevisionHandler ```

Small utility class for executing KnowledgeFlow
flows outside of the KnowledgeFlow application

**Version:**
:   $Revision: 7059 $

**Author:**
:   Mark Hall (mhall{[at]}pentaho{[dot]}org)

---

| **Nested Class Summary** | |
| --- | --- |
| `static class` | `FlowRunner.SimpleLogger` |


**Constructor Summary**

`FlowRunner()`
  
          Constructor


| **Method Summary** | |
| --- | --- |
| `Environment` | `getEnvironment()`             Get the environment variables that are in use. |
| `java.util.Vector` | `getFlows()`             Get the vector holding the flow(s) |
| `java.lang.String` | `getRevision()`             Returns the revision string. |
| `boolean` | `getStartSequentially()`             Gets whether Startable beans will be launched sequentially or all in parallel. |
| `void` | `load(java.lang.String fileName)`             Load a serialized KnowledgeFlow (either binary or xml) |
| `void` | `loadBinary(java.lang.String fileName)`             Load a binary serialized KnowledgeFlow |
| `void` | `loadXML(java.lang.String fileName)`             Load an XML serialized KnowledgeFlow |
| `static void` | `main(java.lang.String[] args)`             Main method for testing this class. |
| `void` | `run()`             Launch all loaded KnowledgeFlow |
| `void` | `setEnvironment(Environment env)`             Set the environment variables to use. |
| `void` | `setFlows(java.util.Vector beans)`             Set the vector holding the flows(s) to run |
| `void` | `setLog(Logger log)` |
| `void` | `setStartSequentially(boolean s)`             Set whether to launch Startable beans one after the other or all in parallel. |
| `void` | `stopAllFlows()` |
| `void` | `waitUntilFinished()`             Waits until all flows have finished executing before returning |

**Methods inherited from class java.lang.Object**

`equals, getClass, hashCode, notify, notifyAll, toString, wait, wait, wait`

**Constructor Detail**

### FlowRunner

```
public FlowRunner()
```

:   Constructor


**Method Detail**

### setLog

```
public void setLog(Logger log)
```

---


### stopAllFlows

```
public void stopAllFlows()
```

---


### waitUntilFinished

```
public void waitUntilFinished()
```

:   Waits until all flows have finished executing before returning

---


### load

```
public void load(java.lang.String fileName)
          throws java.lang.Exception
```

:   Load a serialized KnowledgeFlow (either binary or xml)

    :   **Parameters:**: `fileName` - the name of the file to load from **Throws:**: `java.lang.Exception` - if something goes wrong

---


### loadBinary

```
public void loadBinary(java.lang.String fileName)
                throws java.lang.Exception
```

:   Load a binary serialized KnowledgeFlow

    :   **Parameters:**: `fileName` - the name of the file to load from **Throws:**: `java.lang.Exception` - if something goes wrong

---


### loadXML

```
public void loadXML(java.lang.String fileName)
             throws java.lang.Exception
```

:   Load an XML serialized KnowledgeFlow

    :   **Parameters:**: `fileName` - the name of the file to load from **Throws:**: `java.lang.Exception` - if something goes wrong

---


### getFlows

```
public java.util.Vector getFlows()
```

:   Get the vector holding the flow(s)

    :   **Returns:**: the Vector holding the flow(s)

---


### setFlows

```
public void setFlows(java.util.Vector beans)
```

:   Set the vector holding the flows(s) to run

    :   **Parameters:**: `beans` - the Vector holding the flows to run

---


### setEnvironment

```
public void setEnvironment(Environment env)
```

:   Set the environment variables to use. NOTE: this needs
    to be called BEFORE a load method is invoked to ensure
    that the ${Internal.knowledgeflow.directory} variable get
    set in the supplied Environment object.

    :   **Parameters:**: `env` - the environment variables to use.

---


### getEnvironment

```
public Environment getEnvironment()
```

:   Get the environment variables that are in use.

    :   **Returns:**: the environment variables that are in ues.

---


### setStartSequentially

```
public void setStartSequentially(boolean s)
```

:   Set whether to launch Startable beans one after the other
    or all in parallel.

    :   **Parameters:**: `s` - true if Startable beans are to be launched sequentially

---


### getStartSequentially

```
public boolean getStartSequentially()
```

:   Gets whether Startable beans will be launched sequentially
    or all in parallel.

    :   **Returns:**: true if Startable beans will be launched sequentially

---


### run

```
public void run()
         throws java.lang.Exception
```

:   Launch all loaded KnowledgeFlow

    :   **Throws:**: `java.lang.Exception` - if something goes wrong during execution

---


### main

```
public static void main(java.lang.String[] args)
```

:   Main method for testing this class.

    Usage:  
      

    ```
    Usage:\n\nFlowRunner
    ```

    :   **Parameters:**: `args` - command line arguments

---


### getRevision

```
public java.lang.String getRevision()
```

:   **Description copied from interface: `RevisionHandler`**
:   Returns the revision string.

    :   **Specified by:**: `getRevision` in interface `RevisionHandler`
    :   **Returns:**: the revision


---


|  |  |  |
| --- | --- | --- |
| **Overview** \| **Package** \| **Class** \| **Tree** \| **Deprecated** \| **Index** \| **Help** \| **Weka's home** | |  |
| **PREV CLASS**   **NEXT CLASS** | **FRAMES**    **NO FRAMES**     **All Classes** |  |
| SUMMARY: NESTED \| FIELD \| CONSTR \| METHOD | DETAIL: FIELD \| CONSTR \| METHOD |  |


---
